# Determining Material Removal and Electrode Wear in Electric Discharge Machining with a Generalist Machine Learning Framework

**DOI:** 10.3390/ma19020438

**Published:** 2026-01-22

**Authors:** Jorge M. Cortés-Mendoza, Agnieszka Żyra, Andrei Tchernykh, Horacio González-Vélez

**Affiliations:** 1 Cloud Competency Centre, National College of Ireland, Mayor Street, IFSC, D01 K6W2 Dublin, Ireland; horacio@ncirl.ie; 2 Faculty of Mechanical Engineering, Cracow University of Technology, al. Jana Pawła II 37, 31-864 Cracow, Poland; agnieszka.zyra@pk.edu.pl; 3 Computer Science Department, CICESE Research Center, Carr. Tijuana-Ensenada 3918, Ensenada 22860, BC, Mexico; chernykh@cicese.mx; 4 The Ivannikov Institute for System Programming of the RAS, Alexander Solzhenitsyn st. 25, 109004 Moscow, Russia

**Keywords:** electric discharge machining, material removal rate, electrode wear rate, cryogenic treatment, machine learning

## Abstract

Electric Discharge Machining (EDM) is a well-established process for fabricating complex geometries from hard materials. However, identifying the influence of process parameters remains challenging and costly due to the stochastic nature of EDM and the expense of experimental validation. Machine Learning (ML) techniques provide an alternative to mitigate these limitations by enabling predictive modeling with reduced experimental effort. This research proposes a generalizable framework employing four ML models to analyze the correlation between EDM inputs and outputs, incorporating 11 levels of cryogenic electrode treatment. Independent variables include electrode material, cryogenic conditions, pulse current, and pulse duration, while performance is assessed through Material Removal Rate (MRR) and Electrode Wear Rate (EWR). The results demonstrate that Random Forest (RF) and Artificial Neural Networks (ANNs) achieve superior predictive performance compared to alternative approaches, improving the $R^{2}$ metric from 0.973 to 0.9956 for EWR in the case of an ANN and from 0.980 to 0.9943 for RF with MRR, compared with previous work in the literature and the best methods across 30 executions. Both models consistently yield high predictive accuracy, with $R^{2}$ values ranging from 0.9936 to 0.9979 in training and testing datasets. Furthermore, ANN significantly reduces mean squared error, decreasing EWR prediction error from 5.79 to 0.68 and MRR error from 122.75 to 35.89. This research contributes to a deeper understanding of EDM process dynamics.

## 1. Introduction

Electric Discharge Machining (EDM), also known as spark machining or spark eroding, enables the machining of intricate shapes in conductive materials regardless of their hardness [1,2]. Material removal occurs due to a phenomenon that accompanies pulsed electrical discharges between the working electrode and the workpiece, separated by a dielectric in the machining gap [1,3]. The process avoids physical contact between the working material and the workpiece, where the sufficient voltage difference between them gives rise to a plasma channel of high energy density. The high temperature of the inter-electrode gap melts or ablates the workpiece material [4,5]. The EDM process maintains a forced dielectric flow with essential functions: dissipating heat from the workpiece and the working electrode, removing solidified particles from the machining gap, and stabilising the process conditions, allowing for the occurrence of subsequent discharges [6,7,8]. The thermal nature of this process causes a change in the physical properties of the surface layer, resulting in the characteristic formation of three thermally modified layers [9]. In the first layer, microcracks, which are associated with residual stresses, surface porosity, grain boundary growth, and changes in chemical composition might be observed [10,11]. EDM has been adopted in several branches of industry, such as automotive, aerospace, chemical, aviation, nuclear, petroleum, and medical, because it can be used when conventional machining techniques are not cost-effective [12,13], e.g., when materials are hard to machine—such as titanium alloys and nickel-based superalloys, among others—or to produce small, thin-walled elements with complicated geometry, a.k.a. ”micro-machining” [14]. The input parameters of the EDM process—current intensity, gap voltage, pulse on time, pulse out time, dielectric type, and working electrode material—can substantially affect the quality of the final product [3,15].

In the literature, authors delineate a broad spectrum of methodological approaches and application strategies in which Machine Learning (ML) techniques are employed to predict or optimize key output characteristics of the EDM process. These studies underscore that data-driven models—ranging from classical regression frameworks to advanced deep learning architectures—can be systematically trained to estimate machining performance indicators such as material removal rate, tool electrode wear, and the resulting surface integrity. Numerous contributions highlight that the integration of supervised learning algorithms not only enables the approximation of nonlinear relationships between process parameters and output responses but also supports multi-objective optimization aimed at achieving desired technological outcomes [16,17].

Cetin et al. [5] evaluate several ML algorithms to predict electro-erosion wear in cryogenic-treated electrodes of mold steels. The authors consider algorithms in the domain of Ensemble Learning (EL), ANN, Boosting, Decision Trees (DTs), and K-Nearest Neighbours (KNNs). The inputs of the ML techniques are the Electrode Material (EM), Cryogenic Process Conditions (CPC), Pulse Current (PC), and Pulse Duration (PD). The results show that the EL models provide an accuracy of almost 99% during the training and testing phases, according to a simple split of the dataset and R-squared ($R^{2}$) metric. In addition, they identified the most relevant characteristics that affect wear patterns. Arunadevi and Prakash [7] analyse the MRR and Surface Roughness (SR) to machining Al7075 + 10% Al_2_Ol_3_ materials using WEDM. ANN and LR with pulse time ($T_{on}$), Voltage (Vo), pulse-off ($T_{off}$), Bed Speed (BS), and Current Intensity (CI) are used to predict output parameters and to find input values that maximise MRR and minimise SR. The results show that ANN outperforms LR considering MRR and SR. The authors also identify the optimal solution using a Pareto front. Jatti et al. [10] present several ML models for the prediction of MRR during EDM of Nickel-Titanium (NiTi), Nickel-Copper (NiCu), and Beryllium-Copper (BeCu) alloys. ML regression and classification models based on RF, DTs, Gradient Boosting (GB), ANN, and Adaptive Boosting (AdaBoost) predict MRR and an MRR value below 5 mm^3^/min. The input parameters of the algorithms are Workpiece Electrical Conductivity (WEC), Gap current (Gc), Gap voltage (Gv), $T_{on}$, and $T_{off}$. The results show that GB is the most efficient regression algorithm for predicting MRR with a value $R^{2}$ = 0.930, and RF outperforms other classification algorithms based on the F1-score with a value of 1. ML can accurately predict machining performance, support tool design, and process parameter optimisation. In addition, the authors identify that Gc and Vo have a dominant influence on MRR, and cryo-treated electrodes significantly affected MRR. Kaigude et al. [12] evaluate the prediction of LR, DTs, and RF for SR with AISI D2 steel and EDM and Jatropha oils as dielectric media. The input parameters consider PC, Gv, T_*on*_, and T_*off*_. The results show that RF provides high accuracy prediction with an $R^{2}$ value of 0.89 and an MSE of 1.36%. Bhandare and Dabade [13] propose an ANN for the prediction of MRR, Tool Wear Rate (TWR), and SR considering Gas Dielectric Pressure (GDP), PC, Spark on time (S_*on*_), and Gap Spark Voltage (GSV). The results show the effectiveness of the ANN with a prediction accuracy of 89.09%, 87.30%, and 84.83% for MRR, TWR, and RS, considering the Mean Squared Error (MSE) metric, respectively. Ilani and Banad [14] propose the EDMNet framework with 12 ML approaches based on Deep ANN (DNN), SVR, Voting (VT), Bootstrap aggregating (Bagging), Extreme Bagging (XBagging), AdaBoost, LR, Ridge regression, Lasso regression, and Elastic Net regression to predict EDM performance. The prediction of MRR, EWR, and SR (R_*a*_) considers CI, Scanning Speed (SS), $T_{on}$, Powder Concentration (PwC), Injection Pressure (IP), Vibration Frequency (VF), and Amplitude of Vibration (AV). The results show that EDMNet can be used as a reproducible standardised benchmarking framework that facilitates ML model comparison in the EDM context. The authors demonstrated that Multiple LR (MLR) exhibits notably inferior performance compared to non-linear learners such as ANN and Decision Tree Regression (DTR), highlighting the complexity of the parameter–response mapping. Cortés-Mendoza et al. [18] study four ML approaches to describe the correlation of the input and output of the dry EDM process with distilled water as a coolant for Inconel 625 and Titanium Grade 2. The prediction models based on LR, RF, SVR, and ANN receive independent variables, $T_{on}$, CI, Vo, Gas Pressure (GP), and Workpiece Material (WM) to estimate MRR, EWR, working electrode velocity (U), and SR parameters ($R_{z}$ and $R_{sk}$). The average efficiency of the models according to the best $R^{2}$ values for MRR, EWR, and U was 0.6735, 0.7955, and 0.7739, using a 5-fold Cross-Validation technique (5CV). Ziyad et al. [19] develop algorithms for DT, RF, GB, and Extreme GB (XGB) to predict SR with AISI 1060 steel. The prediction strategies consider cutting speed (Vc), Feed rate (F), workpiece Hardness (H), and Machining Environment (ME). The combination of DT, GB, and XGB generates a Super Learner Model (SLM) that improves the predictive efficiency of the models. Additionally, the interpretations of the model’s predictions are clarified using the Shapley additive explanations (SHAP) technique. Ali et al. [20] address the issue of accurately predicting the MRR, TWR, and SR output parameters in micro-EDM drilling using advanced ML methods. This study aims to develop predictive models for EDM setups using ensemble ML techniques for the TNTZ (Ti-29Nb-13Ta-4.6Zr) machined alloy. An MLR, a DT, and an ANN estimate the output parameters Volume Removal Rate (VRR), Overcut (Ov), Circularity Error (CE), and SR parameters. The evaluation employs two key performance metrics: the Normalised RMSE (NRMSE) and the $R^{2}$ factor. The results show that MLR performed poorly due to the non-linear nature of the data (with the lowest $R^{2}$ and highest NRMSE), DTR showed moderate accuracy (with $R^{2}$ > 75%, and prediction error < 10% in most cases), and ANN delivered the best results (with $R^{2}$ > 99% and prediction errors < 5%) in all outputs for training and testing data. Sarker et al. [21] define a methodology to develop optimised weighted average ensemble models to predict the output parameters of Vc, SR and Spark Gap (SG) in Wire EDM (WEDM) processes. RF, SVR, and Ridge are used as base models, which represent a different modelling paradigm. The two ensemble models aimed to enhance prediction accuracy by minimising the RMSE and Mean Absolute Error (MAE) using weighted averaging of the base models and derived weights. The evaluation considers multiple performance metrics: RMSE, MAE, MSE, R, MAPE, RMSPE, RMSLE, RAE, RRSE, and the Mean Relative Signal-to-Noise (MRSN) ratio to evaluate the prediction of $T_{on}$, $T_{off}$, Spark gap Voltage (SgV), Peak Current (PkC), Wire Tension (WT), and Wire Feed (WF). The results indicated that the RMSE optimised ensemble model achieved the highest prediction accuracy in all response variables, and the MAE optimised ensemble model outperformed individual base models, with a few exceptions. Mandal et al. [22] study the Monel K500 EDM process using a new metaheuristic technique called the Multiobjective Dragonfly (MODA). MODA receives as input PkC, $T_{on}$, Duty Cycle (DC), and Servo Voltage (SV) to predict the parameters of MRR and EWR. The results show that the model predictions reached $R^{2}$ values of 99.40% and 96.60% for MRR and EWR, respectively. Moreover, the methodology considers a Box–Behnken design to prepare the experimental design matrix, and it allows authors to identify a set of non-dominated solutions and the optimal process input parameters. Kumar and Jayswal [23] present an NN to optimise the prediction of MRR with WEDM a synthetically generated dataset. $T_{on}$, $T_{off}$, CI, Vo, WF Rare (WFR) are the inputs of the NN model to estimate MRR. The results show a $R^{2}$ value of 0.9999, a MAE value of 0.0166, and an MSE value of 0.0004 for the best NN model based on the Sigmoid activation function. The authors highlight the near-perfect correlation between the predicted and actual NN values for manufacturers, who can optimise process parameters to achieve desired machining outcomes, thereby enhancing efficiency and reducing costs.

Table 1 summarises the main characteristics and parameters, together with their methodologies and materials, of the related works. The increasing interest in applying ML techniques to model and predict output parameters in EDM arises from the inherent complexity of the process and the non-linear relationships between input and output parameters. Despite promising results in numerous studies, researchers continue to face a range of methodological and technical challenges that constrain the effectiveness and generalisability of ML applications in this field.

In light of these, the present study proposes a generalist ML framework designed to enhance the predictive accuracy and robustness of models describing Material Removal Rate (MRR) and working electrode wear (EWR) when using Copper–Tungsten (CT) electrodes, as originally characterised in [5]. To accomplish this, the research systematically evaluates four predictive modelling approaches—Linear Regression (LR), Random Forest (RF), Support Vector Regression (SVR), and Artificial Neural Networks (ANN)—for forecasting MRR and EWR based on key input parameters, namely Electrical Mode (EM), Capacitance (CPC), Pulse Current (PC), and Pulse Duration (PD). This framework draws inspiration from the methodology first outlined in Authors’ previous work [18]. The primary goal of this research is therefore to establish a versatile and empirically validated ML-based modelling strategy that not only improves predictive performance for MRR and EWR under limited data conditions but also advances the development of more generalisable and transferable EDM prediction models.

## 2. Materials and Methods

### 2.1. Test Stand and Sample

**Figure 1:**
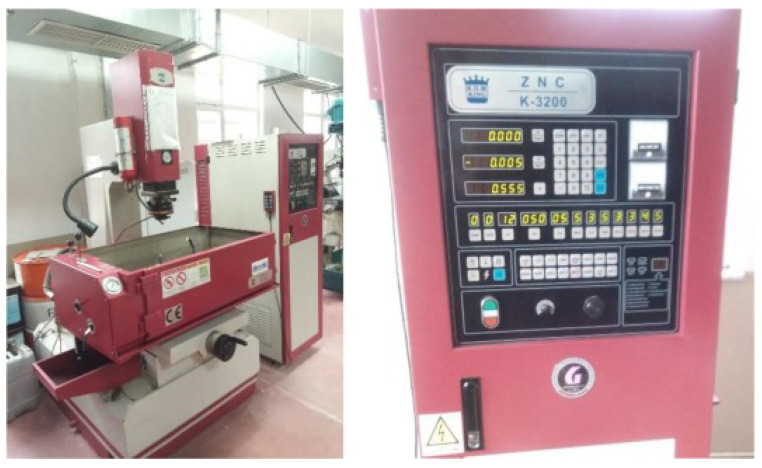
King ZNC K-3200 EDM machine and its control panel of the experimental setup to perform the tests [5].

EDM experiments were performed on a King ZNC K3200 die-sinking EDM machine (Kingred Intelligent Equipment (Suzhou) Co., Ltd., Suzhou, China), see Figure 1. The tests were carried out under constant processing conditions except when deliberately modifying a specific parameter. The dielectric fluid used was Petrofer Dielectricum 358 (PETROFER Chemie, Hildesheim, Germany), a mineral-based oil formulated for electro-erosive machining.

**Figure 2:**
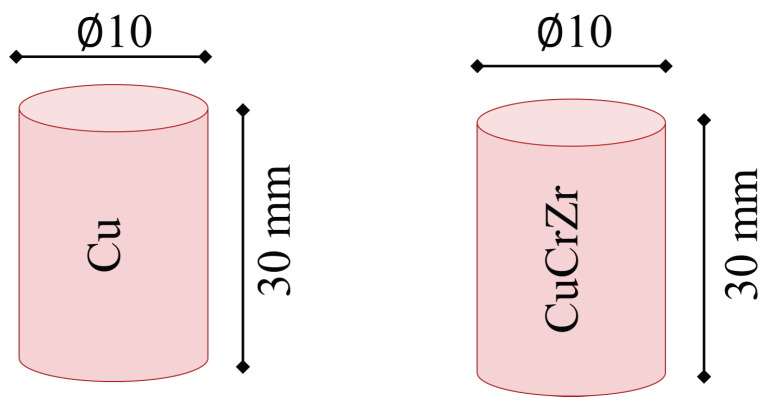
Cu and CuCrZr cylindrical working electrode characteristics of the experimental setup to perform the tests [5].

**Figure 3:**
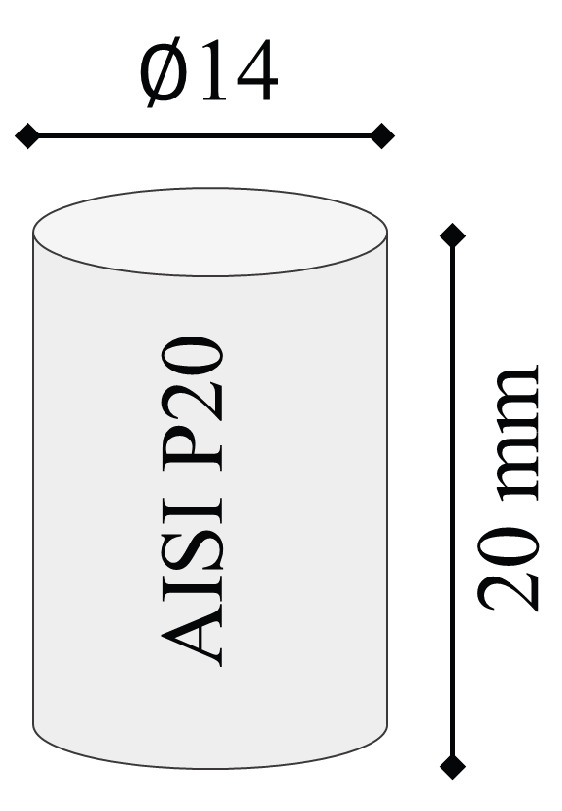
AISI P20 workpiece material characteristics [5].

**Figure 4:**
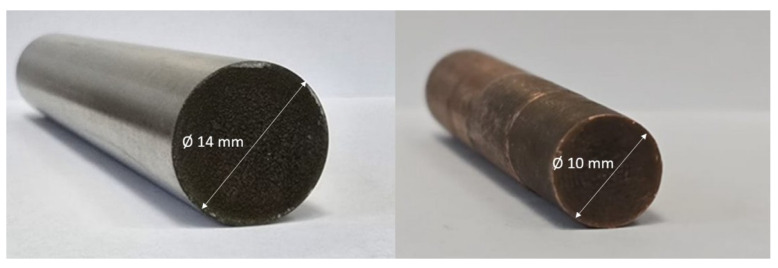
Example of workpiece and working electrode.

Cylindrical working electrodes made from Cu and CuCrZr, with dimentions of 10 × 30 mm dimensions, see Figure 2, were used for EDM. The cylindrical workpiece material made of AISI P20 tool steel, a common material in plastic injection mold applications with dimentions of 14 × 20 mm was machined (Figure 3). Furthermore, Figure 4 shows examples of workpiece material and working electrode, and Table 2 shows both the chemical composition and properties of electrode materials.

### 2.2. Experimental Plan

In total, 176 EDM experiments based on the four-factor test plan with the following variables: Electrode Material (EM), Cryogenic Process Conditions (CPC), Pulse Current (PC), and Pulse Duration (PD), were outperformed by [5]. Table 3 shows the input parameters and the values during the EDM experiments, Section 2.4 provides more detailed information on these values.

The electrodes were divided into 11 levels of CT to assess their impact. The cryogenically treated electrodes underwent treatment cycles at −140 °C for 15 and 30 min, as well as 0, 0.25, 0.5, 1, 2, 4, 8, 12, 16, 20, and 24 h, followed by tempering at 175 °C for one hour [5]. Deep CT in the range of −125 and −196 °C enhances certain mechanical properties of tool steels, and the most significant increase in properties is obtained by CT between quenching and tempering [24]. The literature describes several improvements in the mechanical properties of cryo-treated tool steels: Complete transformation of retained austenite into martensite, precipitation of fine dispersed carbides and removal of residual stresses [25].

Each test was repeated three times, and the average of the three experiments was considered as a test result, to ensure their precision. While EDM is an inherently stochastic and random process; it is not possible to predict the exact location of the first discharge, and thus the point at which dielectric breakdown will occur. It is also a process strongly dependent on voltage, current intensity, and pulse duration, while the material of the tool electrode and the workpiece itself exert a particularly significant influence on the stability of the machining operation. For this reason, performing three repeated trials under identical input conditions provides a necessary level of statistical reliability for the measured outputs. All input parameters of the process were kept constant throughout the experimental campaign, and every test was conducted using the same dielectric fluid. This dielectric—specifically formulated for die-sinking EDM—ensures appropriate dielectric strength, effective heat removal from the machining zone, good arc-quenching capability, high flash point, adequate stability, and chemical compatibility with the machine components. Since EDM is fundamentally a thermally driven process, the literature reports that the temperature within the plasma channel can reach values as high as 14,000 K. Consequently, maintaining constant values of voltage, current, and pulse duration and same workpiece and working electrodes materials—allows us to assume that the repeatability across the individual trials was preserved to a high degree.

The dataset comprises key variables with distinct central tendencies and levels of dispersion. The Cryogenic Process Conditions (CPC) exhibit a mean value of 7.98 with a standard deviation of 8.34, indicating substantial variability relative to the mean. Electrode Wear (EW) presents a mean of 16.47 and a standard deviation of 15.24, highlighting the pronounced variability in tool degradation [5]. Finally, Workpiece Wear (WW) records the highest mean value at 154.65 and a large standard deviation of 82.69, indicating considerable spread and sensitivity in material removal results [5].

Mass losses after the EDM process define the EWR and MRR values for Cu and CuCrZr electrodes, and workpieces. The methodology consists of weighting the difference between the Mass Before Testing (MBT) and the Mass After Testing (MAT) of the electrodes ($MBT_{e}$ and $MAT_{e}$) and the workpieces ($MBT_{w}$ and $MAT_{w}$). Both the tool electrode and the workpiece were weighed using a high-precision analytical balance with an accuracy of 0.0001 g; see Ref. [5].

The EWR and MRR are determined by the following formulas:

(1) $$EWR\left[\mathrm{mg}/\min\right]=\frac{MBT_{e}-MAT_{e}}{T},$$

(2) $$MRR\left[\mathrm{mg}/\min\right]=\frac{MBT_{w}-MAT_{w}}{T},$$

where *T* = 20 is the EDM process time in minutes.

In Table 4, the Pearson Correlation coefficient for set of input and output parameters was presented. Pearson Correlation coefficient represents the linear dependence between pairs of variables in the dataset. Each coefficient reflects both the strength and the direction of the linear relationship, with values ranging from −1 to +1. A coefficient close to +1 indicates a strong positive linear association, whereas a value near −1 reflects a strong negative linear relationship. Coefficients around 0 suggest the absence of a linear dependency, although nonlinear associations may still be present. In the context of this study, the Pearson matrix provides an initial indication of how input parameters—such as current intensity, voltage, and pulse duration—co-vary with output measures such as MRR and EWR. Identifying variables with higher absolute correlation values helps determine which features may exert a more substantial influence on the predictive modelling process, while weaker correlations signal parameters that may contribute less to linear model performance. While analysing the Pearson Correlation coefficient one could notice that working electrode wear and material removal rate are strongly correlated with current intensity (positive correlation) and workpiece wear is correlated with working electrode wear (positive correlation), which means that these parameters have the greatest influence on the EDM process.

### 2.3. Machine Learning Framework

From the authors’ perspective, the central objective of the planned investigations was to examine the feasibility of applying ML techniques to predict tool-electrode wear and to estimate the machining efficiency of the EDM process conducted under various parameter settings and using electrodes subjected to different cryogenic treatment cycles. For this reason, the subsequent sections of the study concentrate on identifying the most effective statistical and ML algorithms capable of modelling process behaviour and forecasting its outcomes—namely, the material removal rate and the tool-electrode wear. These two output variables represent one of the key performance indicators in EDM and are essential for assessing both the stability and effectiveness of the machining process.

Accordingly, the following discussion is devoted directly to methodological aspects related to machine learning, with the aim of determining which approaches provide the most accurate and robust predictive capability for the EDM process under the conditions investigated.

ML focusses on statistical algorithms that can identify patterns in the data and use them to predict [26]. Supervised ML is a category of ML paradigm in which models learn from the labelled datasets to make predictions or classifications on unseen data. They learn the relationship between input and output data. Recently, several (supervised) ML methods have overcome the performance of previous approaches or even human skills in some tasks. Our generalist framework employs four ML methods—LR, RF, SVR, and ANN—to systematically determine the EDM’s EWR and MRR. They are standard methods in the literature for predicting.

Linear Regression (LR). First introduced by Galton in the late nineteenth century [27], LR fits a straight line to the data (“linear equation”) that better represents the relationship between the dependent variable and the independent features [28]. This equation minimises the error between the predicted and actual values with respect to the training dataset via the residual sum of squares, a standard measure to define the level of variance in the error term (“residuals”) of the model [26]. The LR parameters are easier to fit than other non-linear models, and the statistical properties of the resulting estimators are easier to determine. LR is a standard baseline model in ML due to its simplicity, interpretability, and computational efficiency.

Random Forest (RF). Initially used by Morgan and Sonquist in the early 1960s to examine determinants of social conditions by recursive partitioning [29], a Decision Tree (DT) is a non-linear alternative to LR that consists of dividing (“partitioning”) the space into smaller regions where interactions are more manageable [30]. By implementing recursive partitions of the space, DTs generate fragments to create simpler models. A prediction over a tree considers the partitions that define the tree’s structure. The training phase of a DT finds the thresholds or cuts that create the regions which minimise the MSE. Overfitting, high variance, and bias are some important limitations of DTs. To overcome such limitations, Ho [31] proposed to build multiple trees in stochastically selected subspaces of the feature space whose capacity could be arbitrarily expanded for increases in accuracy for both training and unseen data. Known as a Random Forest (RF), it creates several DTs as a single model to generate the prediction, reducing the limitations of a single DT. The random sampling with replacement and the random subset of features for each DT in RF decrease the variance of the model and the correlation between DTs. This ensemble learning algorithm estimates the final output using the mean output values of the DTs in the RF, reducing the tendency of the individual trees’ to overfit the data.

Support Vector Regression (SVR). Proposed by Basak, Pal, and Patranabi [32], SVR implements LR in a high dimensional feature space where input data are mapped via a non-linear function based on the seminal Vapnik–Chervonenkis work [33]. The SVR algorithm establishes a region around the function with a certain tolerance where the points within the region are considered correct predictions [34]. The main goal of the tube region is to find the best approximation of the continuous-valued function so that the predictions inside the tube region minimise the error function. SVR uses Kernel functions to deal with nonlinear processes where original data are projected into high-dimensional feature spaces where linear or more complex relationships may exist. Good performance with multidimensional data, handling small datasets, processing of high-dimensional count datasets, modelling non-linear decision boundaries, and a low resource-hungry algorithm are some advantages of SVR. Some disadvantages include Kernel selection, parameter sensitivity, memory intensive, and lack of probability interpretation.

**Figure 5:**
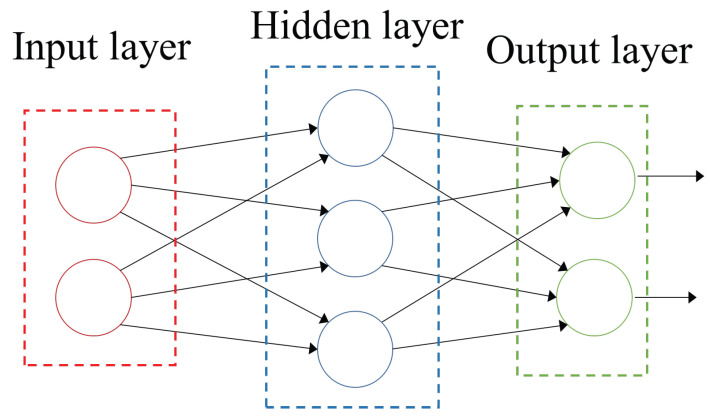
An ANN with an input layer of two neurons (red circles), a hidden layer with three neurons (blue circles), and an output layer with two neurons (green circles), where arrows define the connections and (2-3-2) the configuration of the ANN [18].

Artificial Neural Network (ANN). By systematically deploying Rosenblatt’s Perceptron [35] to learn to classify patterns using adjustable synaptic weights using the McCulloch–Pitts mathematical formalism of a human brain neuron [36], an ANN simulates interconnected neurones that provide the structure to solve complex problems [37]. The network structure consists of independent layers with an arbitrary number of organised neurones. Layers organise interconnected neurones by edges to create the ANN. The three types of layers provide different functionalities: The input layer takes the external signals, the hidden layers process the input information, and the output layer supplies the final result. The training process minimises the error in the output prediction for each neurone in the ANN, considering MSE. Figure 5 shows an example of an ANN with three layers and seven neurones.

An important limitation for developing an ANN is the definition of several values in the structure and training process, such as the number of neurons and layers, the interconnections of the edges, the number of epochs, the activation function, and the learning rate, among others. ANNs are widely used in different domains due to their advantages: no assumptions about data properties or distribution, flexibility, encompassing nonlinear regression models, handling incomplete data and noise, and scalability [38].

Programmed in Python 3.12 with the sklearn 1.4.1 library, our four-method ML framework has been deployed on a workstation with a 10-core Intel(R) Xeon(R) CPU E5-2650 v3 @ 2.30 GHz, 128 GB of memory, and 1.2 TB hard disc, running Linux Ubuntu 20.04.6.

**Figure 6:**
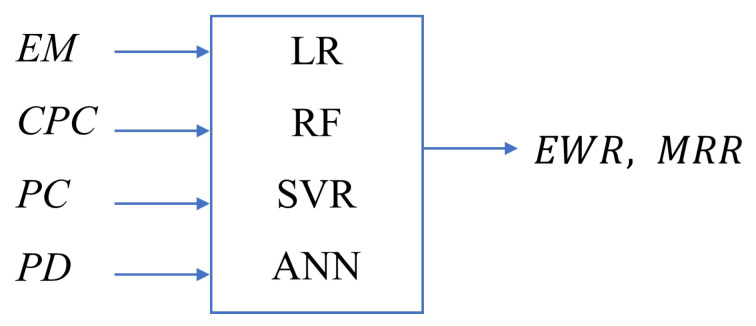
Input and output parameters of the proposed prediction models for the four ML approaches.

The prediction model is trained with the dataset’s information of four inputs and only one output value, EWR or MRR. Figure 6 shows the inputs and outputs of the proposed prediction model for the four ML approaches.

### 2.4. Dataset for Machine Learning

Evaluating strategies with real data is fundamental to measuring their performance. The experimental evaluation considers a dataset with a series of integer and continuous input variables and continuous output values, see [5] for detailed information. Table 5 shows the characteristics of the input values of the dataset. Usually, 24 h of CPC is enough to obtain results [25].

**Figure 7:**
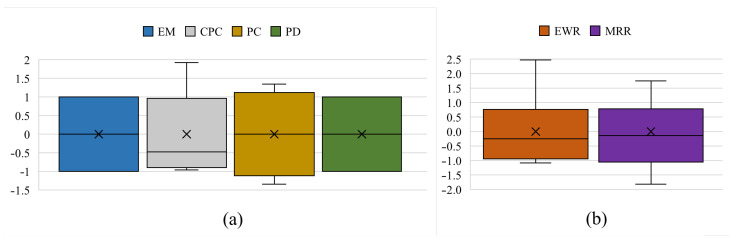
Standardised inputs (**a**) and outputs (**b**) with mean markers (crosses) of the dataset.

All feature values are standardised using a method of feature scaling. The standardisation process centres data around a mean of zero and a standard deviation of one to avoid some features dominating others due to their magnitudes. Figure 7 presents statistical information about the datasets after standardisation, where the standard score of an instance *x* is defined as

(3) z=(x−u)/s,

where *u* and *s* are the mean and standard deviation of the training samples, which allows scaling and scaling back the data between the original representation and its standardizing version.

The Simple Split technique provides a methodology to compare the performance of LR, RF, SVR, and ANN. The dataset is randomly divided into two subsets: 70% for training and 30% for testing. The training dataset is used to train the classification model, and the testing dataset is used to verify the training process. Table 6 shows the number of instances in each training dataset. The training process applies 5CV during the model training phase [39].

### 2.5. Metrics for ML

We consider three standard metrics to evaluate the efficiency of the predictive model: MSE, RMSE, and $R^{2}$. The MSE measures the average of the squares of errors and the RMSE is the quadratic mean of the differences between the observed and predicted values (MSE). Lower MSE and RMSE values indicate that the model predictions are closer to the true values. The statistical measure $R^{2}$ determines the proportion of variance in the dependent variable, which the independent variable can explain. The values of $R^{2}$ close to zero suggest a weak relationship between the variables and indicate a poor fit of the model for the data. MSE, RMSE, and $R^{2}$ are expressed as:

(4) $$MSE=\frac{1}{N}\sum_{i=1}^{N}{\left(y_{i}-{\hat{y}}_{i}\right)}^{2},$$

(5) $$RMSE=\sqrt{\frac{1}{N}\sum_{i=1}^{N}{\left(y_{i}-{\hat{y}}_{i}\right)}^{2}},$$

(6) $$R^{2}=1-\frac{\sum_{i=1}^{N}{\left({\hat{y}}_{i}-y_{i}\right)}^{2}}{\sum_{i=1}^{N}{\left({\bar{y}}_{i}-y_{i}\right)}^{2}},$$

where $y_{i}$ defines the observed value, ${\hat{y}}_{i}$ is the predicted value and ${\bar{y}}_{i}$ specifies the mean value $y_{i}$ for i=1,…,N.

### 2.6. ML Configuration

The configuration of the strategies is fundamental for their performance, as inadequate parameters can limit the efficiency of strategies. So, defining the hyperparameters of strategies is fundamental during the training phase because they control the learning process of the ML model. Hence, the proper hyperparameters improve the prediction model. Hyperparameter optimisation finds values of the parameters that optimise the model by minimising the loss function on given test data.

Hyperparameters are typically chosen empirically, so many models are trained with different configurations on the same training dataset, measuring their performance and retaining the best model [40]. No software in the literature can guarantee that the ideal hyperparameters for the models will be obtained. We used a grid search that exhaustively searches for a predefined set of hyperparameters. Although straightforward and easy to implement, it can be computationally expensive, especially for large hyperparameter spaces [41]. The results showed that the chosen values for the hyperparameters can provide performance similar to that reported in the literature.

Table 7 shows the grid values of the hyperparameters for RF with 5CV using the training dataset. The total number of trained models is 6×6×4×2=288. The hyperparameters for RF include the number of decision trees in the forest (estimators), the maximum depth of the tree (depth), the minimum number of samples required to split an internal node (split), and whether bootstrap samples are used when building trees (bootstrap). The grid search values pursue avoiding overfitting by restricting the depth of the trees, which reduces the arbitrarily complex trees and avoids the memorization of noise, preventing tiny/big subgroups with splitting values that generate simpler trees, and bootstrap sampling helps each tree to see a different sample of the data. Additionally, we validate performance with 5CV to avoid overfitting and underfitting.

Table 8 shows the grid values of the hyperparameters for SVR with 5CV using the training dataset. The total number of trained models is 9×5×4=180. The hyperparameters for SVR are the type of kernel, the degree of the polynomial kernel function, the epsilon value, and the number of iterations.

Table 9 shows the grid values of the hyperparameters for ANN with 5CV using the training dataset and fully connected layers. The total number of trained models is 6336 for 1584 CNNs with one Hidden Layer (HL), 1584 CNNs with two HLs, 1584 CNNs with three HLs, and 1584 CNNs with four HLs. The efficiency of the ANN model depends on the number of HLs and neurons per layer, where the architecture definition follows an empirical procedure that looks for the best hyperparameters. This procedure is expensive in terms of time and computational resources because every combination of hyperparameters must be evaluated.

Appendix A shows the configuration of the best strategies found during the simulation process for the four ML strategies.

## 3. ML Experimental Evaluation

The models with the best performance, considering MSE, RMSE, and $R^{2}$ to predict EWR and MRR, are used to measure their efficiency with unseen data from the testing dataset. This Section presents the values obtained after 30 executions, considering $R^{2}$ as a comparison point with [5], which does not provide standard values for MSE and RMSE. Note that a strategy with the highest value of $R^{2}$ during the testing phase does not necessarily provide the highest value of MSE and RMSE. Moreover, we consider the highest efficiency with respect to unseen data. The analysis of more than one metric can be done using a standard multiobjective analysis, such as the Pareto Front or the weighted sum [7]. In Appendix A, Table A4 shows information about models that consider the MSE and RMSE metrics.

**Figure 8:**
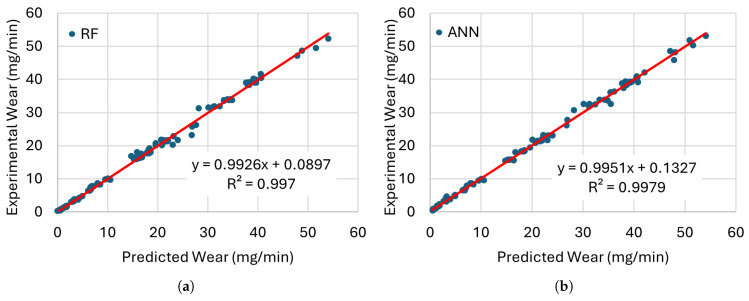
Comparison of the training performance of the best models for EWR with (**a**) RF and (**b**) ANN with their trendlines (red lines).

**Figure 9:**
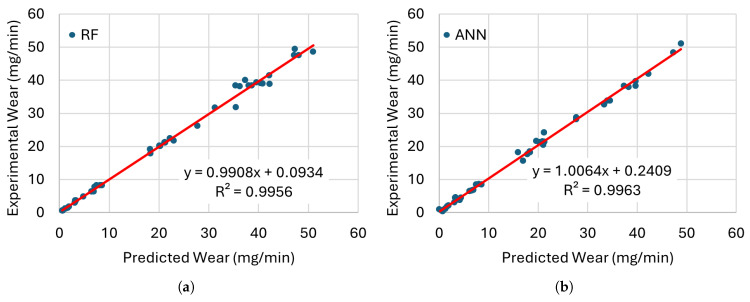
Comparison of the testing performance of the best models for EWR with (**a**) RF and (**b**) ANN with their trendlines (red lines).

**Figure 10:**
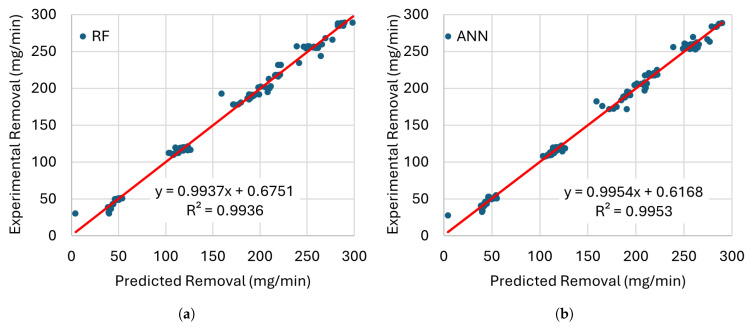
Comparison of the training performance of the best models for MRR with (**a**) RF and (**b**) ANN with their trendlines (red lines).

**Figure 11:**
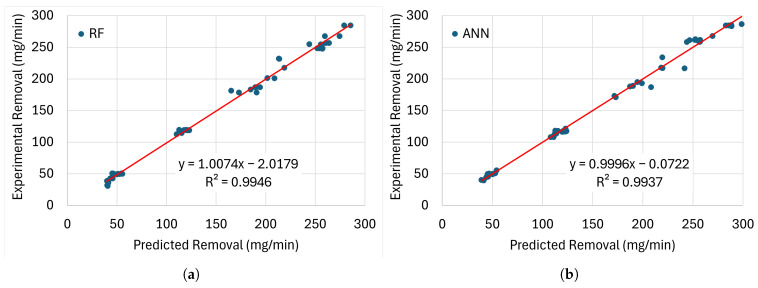
Comparison of the testing performance of the best models for MRR with (**a**) RF and (**b**) ANN with their trendlines (red lines).

Figure 8, Figure 9, Figure 10 and Figure 11 present the error distributions and regression plots corresponding to the training and testing datasets for best methods. The results indicate only minor discrepancies between the experimentally measured and model-predicted values of electrode wear and workpiece material removal. This close agreement suggests that all the investigated models are capable of capturing the underlying relationships between the process input parameters and the resulting EDM performance indicators, thereby providing accurate and reliable predictions within the analysed domain.

Table 10 shows the results of the LR, RF, SVR and ANN performance evaluation with respect to the prediction models. The best value for the four approaches is highlighted in bold font for the two outputs during the training and testing phases. The values represent an average of 30 executions with different seeds after the training and testing processes. The average over a larger number of runs with different seeds enhances the reliability and statistical significance of the results. This approach also helps us to understand the variance and stability of the proposed strategies. It also presents details on the standard deviation of models after 30 executions.

ANN and RF outperform LR and SVR with respect to EWR and MRR. ANN provides the best MSE, RMSE, and $R^{2}$ values for MRR and the EWR training phase. RF performs better than other strategies according to all metrics during the EWR testing phase. In addition, ANN and RF improve the efficiency of state-of-the-art ML algorithms in the literature during the training and testing phases. The $R^{2}$ values of both prediction models are between 0.9808 and 0.9976 for the training and testing phases.

Table 11 shows the percentage of improvement with respect to [5] based on the $R^{2}$ metric. LR only performs worse than the best algorithm in [5] on both outputs and phases, and SVR and ANN with respect to the testing set considering EWR. In other cases, SVR, RF, and ANN improve the efficiency of [5] between 0.13% to 1.77%.

Table 12 shows the results of the LR, RF, SVR and ANN performance evaluation with respect to the prediction models. The best value for the four approaches is highlighted in bold font for the two outputs during the training and testing phases. The values represent the best of 30 executions with different seeds after the training and testing processes, and these results can be directly compared with [5].

ANN and RF outperform LR and SVR with respect to EWR and MRR. ANN provides the best MSE, RMSE, and $R^{2}$ values for the EWR training and testing phases and for the MRR training phase. RF performs better than other strategies considering all metrics for the MRR testing phase. Like the average of 30 executions, ANN and RF improve the efficiency of the latest ML algorithms in the literature during the training and testing phases. The $R^{2}$ values of both prediction models are between 0.9936 and 0.9979 for the training and testing phases.

Table 13 shows the percentage of improvement with respect to [5] based on the $R^{2}$ metric for the best-found strategy. LR only has a lower performance than the best algorithm in [5], except for the testing set of MRR. SVR, RF, and ANN improve the efficiency of [5] between 0.01% and 1.79%.

Furthermore, we analysed MSE and RMSE metrics with the original data to verify the real efficiency of the models. We consider the MSE and RMSE values for the entire dataset. Table 14 shows the MSE and RMSE values for all models with respect to the best predictor of the 30 executions and the best configuration of the model. ANN can reduce the MSE of EWR from 5.79 to 0.68 and the RMSE from 2.41 to 0.83 with respect to the EL models. Similarly, ANN decreases MRR from 122.75 to 35.89 for MSE and from 11.08 to 5.99 for RMSE. In addition, SVR provides better predictions than the more complex models described in [5], which reduces the MSE and RMSE of the EWR from 5.79 to 4.27 and from 2.41 to 2.07, and the MRR values from 122.75 to 78.57 and 11.08 to 8.86. These values proved that our simpler models can outperform the efficiency of more complex models, such as EL, ANN, Boosting, and DT, which simplifies their interpretation and reduces training and evaluation times.

**Figure 12:**
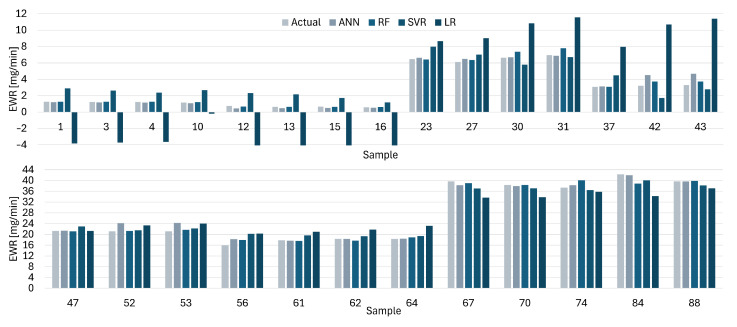
Actual and predicted values of EWR with Cu electrodes for LR, SVR, RF, and ANN.

**Figure 13:**
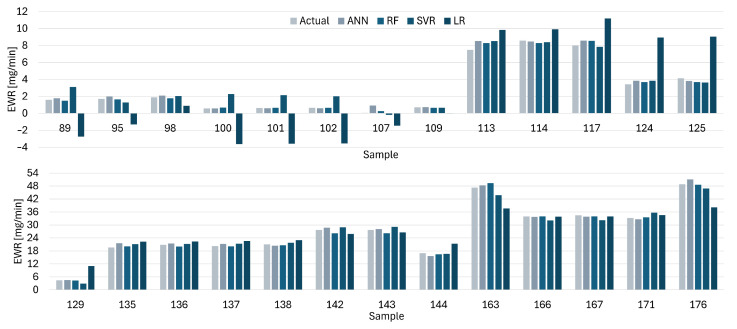
Actual and predicted values of EWR with CuCrZr for LR, SVR, RF, and ANN.

**Figure 14:**
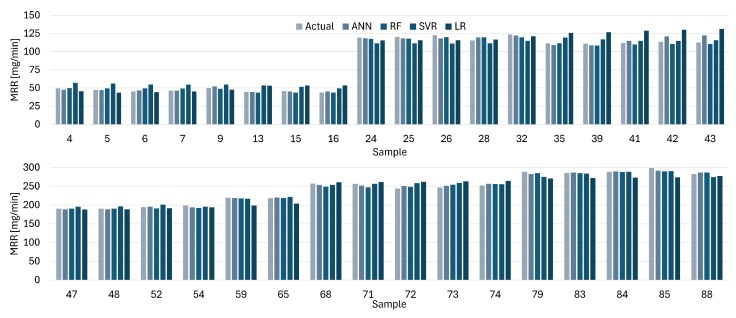
Actual and predicted values of MRR with Cu for LR, SVR, RF, and ANN.

**Figure 15:**
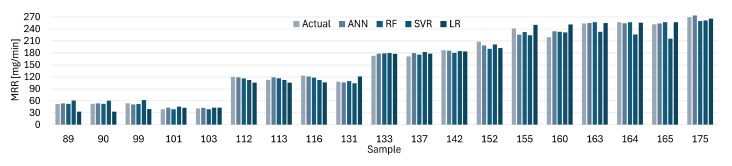
Actual and predicted values of MRR with CuCrZr for LR, SVR, RF, and ANN.

Figure 12, Figure 13, Figure 14 and Figure 15 present comparisons between the expected and predicted values for LR, RF, SVR, and ANN, using all instances from the testing dataset and both electrodes. These prediction models are the best of the 30 trained models with 5CV, see Table 12. In the figures, the sample value defines the index of the instance with respect to the 176 elements in the dataset.

The predicted values for ANN are closer to the actual values for the EWR with the Cu electrode, see Figure 12, which is in line with the results reported for different metrics. LR provides the worst approximation among all the models. The difference between the actual and predicted values is easy to identify for the first 15 samples of the figure. A similar situation is presented for the EWR with the CuCrZu electrode, see Figure 13, where ANN has the best performance and LR the worst.

Similarly, the predicted values for ANN are closer to the actual values for the MRR with the CuCrZu electrode, see Figure 14, according to the reported results for different metrics. LR provides the worst approximation among all the models, but the difference between the actual and predicted values is lower than the results for Cu electrode. Figure 15 shows a similar situation for the MRR with the CuCrZu electrode, where ANN has the best performance and LR the worst.

An important advantage of our strategies and their configuration is the consideration of a limited number of instances in the dataset, a common situation in the field of machining, according to the analysis of related work (see Table 1). Small datasets tend to cause problems of overfitting due to the small data scale and too low feature dimensions. From the ML perspective, the SVR and RF models have been shown to be suitable for prediction with small datasets [42].

Moreover, the configurations of the models limit their flexibility; hence, they avoid overfitting. For instance, the configurations reduce the possibility of overfitting by restricting the depth and complexity of DTs in RF, and limit model complexity and epochs of ANNs. In addition, our methodology rigorously validates the performance by executing several iterations with different seeds and 5CV. It provides a balance between the model’s ability to learn patterns and not memorise noise.

For example, Jatti et al. [10] used 2000 epochs to train the ANN model, but overfitting appeared after 500 epochs, and Kumar [23] reached loss-minimum values around epoch 100, even if the training process considered 1000 epochs. In our implementation, the number of epochs for the best ANN models is 250 for EWR and 150 for MRR, which are lower than the 600 epochs (maximum) in the grid search. In the case of EWR, the $R^{2}$ values are the same between 250 and 600 epochs for the training and testing datasets, which means that the models did not learn more after 250 epochs. In the case of MRR, the $R^{2}$ value is higher after 150 epochs for the training dataset, but lower for the testing dataset, which reflects that the model could incorporate noise.

Underfitting is a possible consequence of limiting the flexibility of the models and the training time. The results and standard deviations of the best models found confirm the absence of underfitting because the values $R^{2}$, MSE, and RMSE are at the same level as the state-of-the-art models, and their standard deviations are between 0.0006 and 0.0485, see Table 10. We consider that the use of grid search with the configurations for small datasets can provide models with high performance and low variability. In addition, training and testing times were reduced due to the low complexity of the models.

Our study has limitations to consider despite the competitive results and evidence of the potential use of our approach. The reduced number of instances in the dataset and a dataset with information of a single EDM machine can limit the interpretability and generality of our findings. However, it provides guidance to EDM users to define the proper configurations of the EDM process without performing preliminary studies. Moreover, it is easily extended to different datasets, configurations, and ML models.

## 4. Conclusions

Traditional experimental or statistical modelling techniques often prove inadequate due to the nonlinear and stochastic nature of EDM processes, particularly when different electrode and workpiece materials are used [16,17]. However, there are still no universal Machine Learning models developed to predict universal EDM output parameters for industrial applications. The EDM process is governed by highly nonlinear, multi-physics phenomena—including dielectric breakdown, plasma channel formation, transient heat transfer, and melt ejection—which are intrinsically stochastic and sensitive to microscopic variations at the electrode–workpiece interface. As shown in numerous studies, the location and intensity of individual discharges vary from pulse to pulse, even under nominally identical parameter settings, which prevents stable, deterministic input–output mappings [43]. This stochastic nature substantially complicates the development of universally transferable Machine Learning models. Moreover, there is the lack of large, standardised, and heterogeneous datasets. EDM experiments are inherently time- and cost-intensive, often restricted to small sample sizes collected under narrow parameter ranges and specific material–tool combinations. Such datasets are not only insufficient for training robust models—particularly deep learning architectures—but also rarely transferable across studies because of differences in electrode materials, workpiece alloys, dielectric fluids, flushing conditions, and machine-specific control strategies. It is also important to underline that EDM performance indicators such as material removal rate and working electrode wear are strongly material-dependent. The thermal and electrical properties of workpiece materials and electrode materials significantly influence discharge behaviour, crater morphology, and wear mechanisms. Consequently, ML models trained on one material–tool–dielectric combination cannot be generalised to other conditions without severe degradation in predictive accuracy. Additionally, variations in dielectric contamination, debris concentration, tool wear progression, and servo-control strategies introduce further sources of non-stationary behaviour not captured in typical datasets. These factors collectively introduce substantial domain-specific biases, making universal EDM prediction models infeasible with current data availability and modelling paradigms.

In our study, we first aimed to identify the machine learning models that are most capable of accurately predicting the output parameters of the EDM process for the specific conditions investigated. Furthermore, through this experimental work, we contribute to expanding the dataset available for improving and refining ML models in this domain. The use of cryogenically treated electrodes has emerged as an alternative to minimising the wear of the working electrode, improving the precision of the machining and enhancing surface quality. However, determining the proper input parameters in the EDM to obtain the required results is complex due to the stochastic nature of the electrical discharges, time-consuming experimentation, and cost.

The prediction models developed considered realistic input parameters such as electrode material, cryogenic conditions, pulse current, and pulse duration. The 5-fold cross-validation (5CV) shows that the average efficiency of Artificial Neural Networks (ANNs) and Random Forest (RF) outperforms Linear Regression and Support Vector Regressor in terms of EWR and MRR. ANN and RF improve the efficiency of the state-of-the-art ML algorithms during the training and testing phases. Their coefficients of determination $R^{2}$ are between 0.9808 and 0.9976 for 30 executions with 5CV, with a $R^{2}$ between 0.9936 and 0.9979 for training and testing with the best predictor of the 30 executions. However, their mean squared errors are 0.84 and 0.68 for EWR, and 41.48 and 35.89 for MMR, which outperform 5.79 and 122.75 described in [5], respectively. The obtained results demonstrate that the proposed approaches achieve higher predictive accuracy than the most effective ensemble-based strategies reported in the literature [5], indicating an improved ability of the developed models to represent the EDM process output parameters. The consistently stable performance observed across a substantial number of independent runs with different random seeds confirms the robustness and reproducibility of the proposed models. This repeatable behaviour provides evidence of their statistical reliability. Consequently, the developed models have the potential to significantly reduce the need for extensive preliminary experimental trials, thereby shortening process setup times and contributing to a reduction in overall manufacturing costs.

However, further studies are necessary to evaluate the actual performance and effectiveness of these models in different operational environments. Future work will focus on incorporating advanced preprocessing techniques and feature engineering strategies to improve the prediction of machining output parameters, extending the analysis to a broader range of workpiece materials, tool electrodes, and developing a comprehensive framework that integrates multiple machine learning approaches.

## Figures and Tables

**Table 1:** Main characteristics of related works in the literature.

ML Model(s)	Machine Type	Inputs	Outputs	Metrics	Material	Dielectric Type	Working Electrode	No. of Trials	Dataset Split	Ref.
EL, Boosting, ANN, DTs, kNN	King ZNC K3200	EM, CPC, PC, PD	MRR, EWR	$R^{2}$, MSE, RMSE, MAPE	AISI P20 tool steel	Petrofer dielectricum 358	Cu, CuCrZr	176	Simple 70-30	[5]
ANN, LR	DK-7732 WEDM	$T_{on}$, $T_{off}$, Vo, BS, CI	MRR, SR	$R^{2}$, residuals, Pareto	Al7075 + 10% Al_2_O_3_	Deionised water	★	18	Simple 90-10	[7]
RF, DTs, GB, ANN, AdaBoost	Electronica Machine Tool C400 × 250	WEC, Gc, Gv, $T_{on}$, $T_{off}$	MRR	MSE, MAE, $R^{2}$, F1-Score, AUC	NiTi, NiCu, and BeCu alloys	Oil with side-wise flushing	Electrolytic Cu	18	Simple 80-20	[10]
LR, DTs, RF	Electronica Machine Tool C400 × 250	PC, Gv, $T_{on}$, $T_{off}$	SR	$R^{2}$, MAE, MAPE, RMSE	AISI D2 steel	EDM and Jatropha oils	Cu	20	CV 66-33	[12]
ANN	CNC EDM	GDP, PC, $S_{on}$, GSV	MRR, TRW, SR	MSE	Inconel 718	Air	Cu	27	Simple 60-40	[13]
DNN, VT, SVR, Bagging, XBagging, AdaBoost, LR, Ridge, Lasso, Elastic Net	Die sinking EDM–501–(50A) spark machine	CI, SS, $T_{on}$, PwC, IP, VF, AV	MRR, EWR, $R_{a}$	MAE, MSE, RMSE, $R^{2}$	Various	Liquid dielectric with powder	★	284	5CV 80-20	[14]
LR, RF, SVR, ANN	Electro-discharge generator MATRIX MPS	$T_{on}$, CI, Vo, GP, WP	MRR, EWR, U, $R_{z}$, $R_{sk}$	$R^{2}$, MSE, RMSE	Inconel 625, Titanium Grade 2	Carbon dioxide with deionised water	Cu	50	5CV 80-20	[18]
DT, RF, GB, XGB, SLM	Powered center lathe CS6266 model	Vc, F, H, ME	SR	$R^{2}$, SME, AME, RMSE	AISI 1060 steel	Biodegradable hydraulic cutting oil	WC and Co with TiCN coating material	48	10CV 80-20	[19]
MLR, DTR, ANN	DT-110i Hybrid μ-EDM	VRR, Ov, CE, SR	MRR, TWR, SR	NRMSE, $R^{2}$	TNTZ alloy	EDM oil	Tungsten carbide	27	Simple 80-20	[20]
RF, SVR, Ridge	CNC-WEDM	$T_{on}$, $T_{off}$, SV, PkC, WT, WF	Vc, SR, SG	RMSE, MAE, MSE, R, MAPE, RMSPE, RMSLE, RAE, RRSE	Stir cast Al/SiCp metal	Deionized water	Brass wire	54	Simple 80-20	[21]
MODA	ELTECH D-300ZNC	PkC, $T_{on}$, DC, SV	MRR, EWR	$R^{2}$	Monel K500	Surfactants + dielectric fluid	Cooper	27	★	[22]
NN	★	$T_{on}$, $T_{off}$, CI, Vo, WFR	MRR	$R^{2}$, MAR, MSE	★	★	★	100	5CV 80-20	[23]
LR, RF, SVR, ANN	King ZNC K3200	EM, CPC, PC, PD	MRR, EWR	*R*^2^, MSE, RMSE	AISI P20 tool steel	Petrofer dielectricum 358	Cu, CuCrZr	176	5CV 70-30	This work

★ N.B. Parameter not explicitly mentioned in referenced paper.

**Table 2:** Chemical composition and properties of electrode materials (wt.%) [5].

	Element (%)	Cu	Cr	Zr
Material	Cu	100	-	-
	CuCrZr	Balance	1	0.1

**Table 3:** Variable input parameters of the experimental plan [5].

Symbol	Parameter	Values
EM	Electrode Material	Cu, CuCrZr
CPC	Cryogenic Process Conditions	0–24 h
PC	Pulse Current	4, 8, 12, 16 A
PD	Pulse Duration	25, 50 μs

**Table 4:** Pearson Correlation coefficients for input and output parameters (*p*-values).

	*p*-Values				
Process Conditions (PC)	1	0	0	0.09	0.02
Current Intensity (CI)	0	1	0	0.94	0.98
Pulse Duration (PD)	0	0	1	−0.06	0.05
Working Electrode Wear (WEW)	0.09	0.94	−0.06	1	0.93
Workpiece Wear (WW)	0.02	0.98	0.05	0.93	1
	PC	CI	PD	WEW	WW

**Table 5:** Input values of the dataset.

Input	Values
EM	{CuCrZr, Cu}
CPC	{0, 0.25, 0.5, 1, 2, 4, 8, 12, 16, 20, 24}
PC	{4, 8, 12, 16}
PD	{25, 50}

**Table 6:** Number of instances in the training and testing datasets after the preprocessing stage.

Inputs	*N*	Training		Testing	
		***EWR***	***MRR***	***EWR***	***MRR***
EM, CPC, PC, PD	176	123	123	53	53

**Table 7:** Parameters of RF to find the best prediction model using 5CV [18].

Parameter	Values	Configurations
Estimators	{50, 100, 150, 200, 250, 300}	6
Depth	{2, 3, 4, 5, 6, 7}	6
Split	{2, 3, 4, 5}	4
Bootstrap	{True, False}	2

**Table 8:** Parameters of SVR to find the best prediction model using 5CV [18].

Parameter	Values	Configurations
Kernel	{linear, polynomial, rbf, sigmoid}	3+6=9 *
Degree	{2, 3, 4, 5, 6, 7}	
Epsilon	{0.1, 0.2, 0.3, 0.4, 0.5}	5
Iterations	{50, 100, 150, 200}	4

* Degree is only applied with a linear kernel.

**Table 9:** Parameters of ANN to find the best prediction model using 5CV [18].

Parameter	Values	Configurations
Epochs	{100, 150, 200, 250, 300, 350, 400, 450, 500, 550, 600}	11
1 hidden layer (1HL)	{10, 20, 30, 40, 50, 60, 70, 80, 90, 100, 110, 120}	12
2 hidden layers (1HL-2HL)	{10-5, 20-10, 30-15, 40-20, 50-25, 60-30, 70-35, 80-40, 90-45, 100-50, 110-55, 120-60}	12
3 hidden layers (1HL-2HL-3HL)	{5-10-5, 10-20-10, 15-30-15, 20-40-20, 25-50-25, 30-60-30, 35-70-35, 40-80-40, 45-90-45, 50-100-50, 55-110-55, 60-120-60}	12
4 hidden layers (1HL-2HL-3HL-4HL)	{5-10-10-5, 10-20-20-10, 15-30-30-15, 20-40-40-20, 25-50-50-25, 30-60-60-30, 35-70-70-35, 40-80-80-40, 45-90-90-45, 50-100-100-50, 55-110-110-55, 60-120-120-60}	12
Activation function	{Hyperbolic tangent (tanh), Logistic, Rectified Linear Unit (ReLU)}	3
Learning rate	{0.05, 0.01, 0.005, 0.001}	4

**Table 10:** Average performance of the best configuration for LR, RF, SVR, and ANN predictors after 30 executions with 5CV according to $R^{2}$.

Output		*EWR*		*MRR*	
Phase		Train	Test	Train	Test
LR	*MSE*	0.0991 ± 0.0073	0.1090 ± 0.0190	0.0257 ± 0.0017	0.0270± 0.0191
	*RMSE*	0.3146 ± 0.0118	0.3290 ± 0.0283	0.1603 ± 0.0055	0.1637 ± 0.1383
	$R^{2}$	0.9011 ± 0.0055	0.8881 ± 0.0166	0.9744 ± 0.0020	0.9719 ± 0.9795
SVR	*MSE*	0.0182 ± 0.0039	0.0351 ± 0.0150	0.0108 ± 0.0011	0.0182 ± 0.0087
	*RMSE*	0.1342 ± 0.0140	0.1830 ± 0.0393	0.1036 ± 0.0053	0.1332 ± 0.0934
	$R^{2}$	0.9818 ± 0.0040	0.9646 ± 0.0133	0.9893 ± 0.0011	0.9813 ± 0.9896
RF	*MSE*	0.0035 ± 0.0010	0.0191 ± 0.0159	0.0052 ± 0.0010	0.0120 ± 0.0061
	*RMSE*	0.0583 ± 0.0084	0.1294 ± 0.0485	0.0716 ± 0.0073	0.1085 ±0.0780
	$R^{2}$	0.9967 ± 0.0010	**0.9808 ± 0.0146**	0.9948 ± 0.0011	0.9875 ± 0.9927
ANN	*MSE*	0.0024 ± 0.0006	0.0229 ± 0.0144	0.0040 ± 0.0008	0.0119 ± 0.0066
	*RMSE*	0.0483 ± 0.0058	0.1441 ± 0.0467	0.0627 ± 0.0066	0.1077 ± 0.0810
	$R^{2}$	**0.9976 ± 0.0006**	0.9771 ± 0.0128	**0.9960 ± 0.0008**	**0.9877 ± 0.9922**
[5]	$R^{2}$	0.980	0.973	0.990	0.980

The best values for the two outputs and two stages are highlighted in bold font.

**Table 11:** Improvement percentage of the models with respect to [5] considering the average value of $R^{2}$ after 30 executions.

Output	Phase	LR	SVR	RF	ANN
*EWR*	Train	−7.89	0.18	1.67	1.76
	Test	−9.19	−1.54	0.08	−0.29
*MRR*	Train	−0.56	0.93	1.48	1.6
	Test	−0.81	0.13	0.75	0.77

**Table 12:** The best performance of LR, RF, SVR, and ANN predictors after 30 executions with 5CV according to $R^{2}$.

Output	Phase	LR			SVR			RF			ANN			[5]
		***MSE***	***RMSE***	$R^{2}$	***MSE***	***RMSE***	$R^{2}$	***MSE***	***RMSE***	$R^{2}$	***MSE***	***RMSE***	$R^{2}$	$R^{2}$
*EWR*	Train	0.1110	0.3331	0.8915	0.0203	0.1425	0.9801	0.0028	0.0532	0.9969	0.0021	0.0461	**0.9979**	0.980
	Test	0.0799	0.2826	0.9149	0.0130	0.1142	0.9861	0.0056	0.0747	0.9954	0.0040	0.0632	**0.9956**	0.973
*MRR*	Train	0.0287	0.1695	0.9710	0.0128	0.1130	0.9879	0.0061	0.0783	0.9936	0.0046	0.0675	**0.9953**	0.990
	Test	0.0194	0.1394	0.9804	0.0087	0.0934	0.9896	0.0062	0.0789	**0.9943**	0.0068	0.0823	0.9936	0.980

The best values for the two outputs and two stages are highlighted in bold font.

**Table 13:** Improvement percentage of the models with respect to [5] considering the best value of $R^{2}$ after 30 executions.

Output	Phase	LR	SVR	RF	ANN
*EWR*	Train	−8.85	0.01	1.69	1.79
	Test	−6.51	0.61	1.54	1.56
*MRR*	Train	−0.9	0.79	1.36	1.53
	Test	0.04	0.96	1.43	1.36

**Table 14:** Performance of the best configuration for LR, RF, SVR, and ANN predictors after 30 executions with 5CV and actual *MSE* and textitRMSE values.

Output	LR		SVR		RF		ANN		[5]	
	***MSE***	***RMSE***	***MSE***	***RMSE***	***MSE***	***RMSE***	***MSE***	***RMSE***	***MSE***	***RMSE***
*EWR*	23.47	4.84	4.27	2.07	0.84	0.92	**0.68**	**0.83**	5.79	2.41
*MRR*	178.33	13.35	78.57	8.86	41.48	6.44	**35.89**	**5.99**	122.75	11.08

The lowest values for the two outputs and two metrics are highlighted in bold font.

## Data Availability

The original contributions presented in this study are included in the article. Further inquiries can be directed to the corresponding author.

## Abbreviations

The following abbreviations are used in this manuscript:

5CV	5-fold Cross-Validation	MRSE	Mean Relative Signal-to-Noise
AdaBoost	Adaptative Boosting	MSE	Mean Squared Error
ANN	Artificial Neural Networks	Ov	Overcut
AV	Amplitude of Vibration	PC	Pulse Current
Bagging	Bootstrap aggregating	PD	Pulse Duration
BS	Bed Speed	PkC	Peak Current
CE	Circulatory Error	PwC	Powder Concentration
CI	Current Intensity	$R_{a}$, $R_{z}$, $R_{sk}$	Surface roughness parameters
CPC	Cryogenic Process Conditions	$R^{2}$	R squared
CT	Cryogenic Treatment	RF	Random Forest
DC	Duty Cycle	Ridge	Ridge Regression
DNN	Deep Neural Network	RMSE	Root Mean Squared Error
DTs	Decision Trees	SG	Spark Gap
EDM	Electric Discharge Machining	SHAP	Shapley additive explanations
			technique
EL	Ensemble Learning	SLM	Super Learner Model
Elastic Net	Elastic Net Regression	Son	Spark on time
EM	Electrode Material	SR	Surface Roughness
EWR	Electrode Wear Rate	SS	Scanning Speed
F	Feed rate	SgV	Spark gap Voltage
GB	Gradient Boosting	SV	Servo Voltage
Gc	Gap current	SVR	Support Vector Regression
GDP	Gas Dielectric Pressure	$T_{off}$	Pulse-off
GP	Gas Pressure	$T_{on}$	Pulse time
GSV	Gap Spark Voltage	TWR	Tool Wear Rate
Gv	Gap voltage	U	Working electrode velocity
H	Workpiece Hardness	Vc	Cutting speed
HL	Hidden Layer	VF	Vibration Frequency
IP	Injection Pressure	Vo	Voltage
KNN	K-Nearest Neighbours	VRR	Volume Removal Rate
Lasso	Lasso Regression	VT	Voting
LR	Linear Regression	WEC	Workpiece Electrical Conductivity
MAE	Mean Absolute Error	WEDM	Wire EDM
MAT	Mass After Testing	WF	Wire Feed
MBT	Mass Before Testing	WFR	Wire Feed Rate
ME	Machining Environment	WM	Workpiece Material
ML	Machine Learning	WT	Wire Tension
MODA	Multiobjective Dragonfly	Xbagging	Extrem Bootstrap aggregating
MRR	Material Removal Rate	XGB	Extreme Gradient Boosting

## Appendix A

The LR models for correlating the process inputs to outputs with standardized values are:

(A1) $$EWR=0.03561338x_{1}+0.09912826x_{2}+0.91865366x_{3}-0.02909239x_{4}$$

(A2) $$MRR=-0.06223156x_{1}+0.02876813x_{2}+0.98352207_{3}+0.06044838x_{4}$$

**Table A1:** Best strategies for Random Forest.

Parameters	Configuration			
	Estimator	Depth	Split	Bootstrap
*EWR*	200	6	2	True/False
*MRR*	200	5	4	True/False

**Table A2:** Best strategies for Support Vector Regression.

Parameters	Configuration			
	Iterations	Epsilon	Kernel	Degree
*EWR*	200	0.02	rbf	-
*MRR*	200	0.02	rbf	-

**Table A3:** Best strategies for ANN.

Parameters	Configuration			
	Epochs	Neurons per Hidden Layers	Activation Function	Learning Rate
*EWR*	250	(60, 120, 120, 60)	ReLU	0.05
*MRR*	150	(55, 110, 110, 55)	ReLU	0.05

**Table A4:** Average performance of the best configuration for LR, RF, SVR, and ANN predictors after 30 executions with 5CV according to MSE and RMSE.

Output	Phase	LR			SVR			RF			ANN		
		*MSE*	*RMSE*	$R^{2}$	*MSE*	*RMSE*	$R^{2}$	*MSE*	*RMSE*	$R^{2}$	*MSE*	*RMSE*	$R^{2}$
*EWR*	Train	0.1110	0.3331	0.8915	0.0211	0.1451	0.9805	0.0028	0.0532	0.9969	0.0027	0.0520	0.9975
	Test	0.0799	0.2826	0.9149	0.0125	0.1119	0.9835	0.0056	0.0747	0.9954	0.0035	0.0595	0.9953
*MRR*	Train	0.0293	0.1712	0.9715	0.0128	0.1130	0.9879	0.0061	0.0782	0.9943	0.0047	0.0688	0.9955
	Test	0.0191	0.1383	0.9795	0.0087	0.0934	0.9896	0.0061	0.0780	0.9927	0.0066	0.0810	0.9922
